# Basomedial amygdala mediates top–down control of anxiety and fear

**DOI:** 10.1038/nature15698

**Published:** 2015-11-04

**Authors:** Avishek Adhikari, Talia N. Lerner, Joel Finkelstein, Sally Pak, Joshua H. Jennings, Thomas J. Davidson, Emily Ferenczi, Lisa A. Gunaydin, Julie J. Mirzabekov, Li Ye, Sung-Yon Kim, Anna Lei, Karl Deisseroth

**Affiliations:** 1 Department of Bioengineering, Stanford University, Stanford, California 94305, USA; 2 CNC Program, Stanford University, Stanford, California 94304, USA; 3 Neurosciences Program, Stanford University, Stanford, California 94305, USA; 4 Department of Psychiatry and Behavioral Sciences, Stanford University, Stanford, California 94305, USA; 5 Howard Hughes Medical Institute, Stanford University, Stanford, California 94305, USA

## Abstract

Anxiety-related conditions are among the most difficult neuropsychiatric diseases to treat pharmacologically, but respond to cognitive therapies. There has therefore been interest in identifying relevant top-down pathways from cognitive control regions in medial prefrontal cortex (mPFC). Identification of such pathways could contribute to our understanding of the cognitive regulation of affect, and provide pathways for intervention. Previous studies have suggested that dorsal and ventral mPFC subregions exert opposing effects on fear, as do subregions of other structures. However, precise causal targets for top-down connections among these diverse possibilities have not been established. Here we show that the basomedial amygdala (BMA) represents the major target of ventral mPFC in amygdala in mice. Moreover, BMA neurons differentiate safe and aversive environments, and BMA activation decreases fear-related freezing and high-anxiety states. Lastly, we show that the ventral mPFC–BMA projection implements top-down control of anxiety state and learned freezing, both at baseline and in stress-induced anxiety, defining a broadly relevant new top-down behavioural regulation pathway.

Human^1,2^ and animal^3,4^ studies have implicated diverse cortical and subcortical regions in anxiety and fear regulation. Interestingly, altered structure^1^ and activity correlations^2^ between mPFC and amygdala have been reported in patients with anxiety disorders, although the precise causal connections remain unclear^3,5^. Complexity is suspected, since ventral and dorsal mPFC (vmPFC and dmPFC, respectively) may have opposing roles in fear (vmPFC inhibits dmPFC^6^, and stimulation of vmPFC^7^ or dmPFC^3^ respectively decreases or increases freezing). Relevant subcortical regions are also complex; inhibitory intercalated cells (ITCs) in amygdala have been hypothesized to be vmPFC targets^8^, and to inhibit fear-promoting cells of the central nucleus of the amygdala, which could be relevant to the decreased freezing caused by electrical stimulation of vmPFC^3^. In contrast, dmPFC innervates the basolateral amygdala (BLA)^9^, and the bulk of the BLA population promotes fear^10–12^. This model could explain vmPFC–dmPFC functional differences^3,10^ and why lesioning ITCs promotes freezing^13^, but has never directly and precisely been tested.

In rats the vmPFC–ITC projection is sparse^8^, suggesting consideration of other targets of mPFC in amygdala for mediation of top-down control. Moreover, prior investigations of mPFC–amygdala circuitry employed electrical^13–15^ or optical stimulation that did not precisely resolve projections defined by cortical subregion origin and amygdala subregion target^7,16,17^. Finally, slower-timescale vmPFC^18–21^ and dmPFC^18,22,23^ lesions and inactivations have generated conflicting effects on anxiety. The precise identity of a functional top-down anxiolytic circuit has thus remained unknown. Here, we resolved distinct mPFC–amygdala projections in mice by combining anatomical tracing, CLARITY, *in vivo* and *in vitro* mapping of functional connectivity, and optogenetic control of mPFC–amygdala projections during fear- and anxiety-related behaviours.

## Direct top-down control of anxiety

**Extended Data Figure 1:**
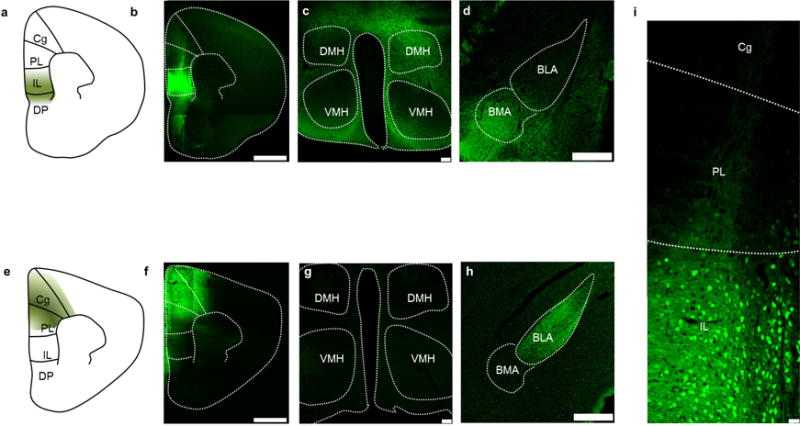
Methodology necessary for targeting viral infusions restricted to vmPFC and dmPFC **a**, Scheme showing subregions of the mPFC. **b**, Representative example from one mouse showing a coronal section containing the vmPFC (IL+ DP) in a mouse expressing YFP in the vmPFC. **c**, **d**, Coronal section depicting vmPFC fibres in the hypothalamus (**c**) and amygdala (**d**). *n* = example from 1 mouse chosen from *n* = 7 mice (**a–d**). **e–h**, Same as **a–d**, but for an animal that received viral injection in the dmPFC (PL and Cg). *n* = example from 1 mouse chosen from *n* = 7 mice (**e–h**). **c**, **g**, Note the presence of fibres from the vmPFC, but not dmPFC, in the hypothalamus. Specifically, the vmPFC projects strongly to the dorsomedial, but not ventromedial hypothalamus (DMH and VMH, respectively). **g**, Section showing expression of YFP in a vmPFC:YFP mouse. *n* = example from 1 mouse chosen from *n* = 7 mice. Cg, cingulate cortex; PL, prelimbic cortex; IL, infralimbic cortex; D P, dorsal peduncular cortex. Scale bars, 1 mm (**b**, **f**); 100 μm (**c**, **g**); 500 μm (**d**, **h**); 35 μm (**i**).

**Extended Data Figure 2:**
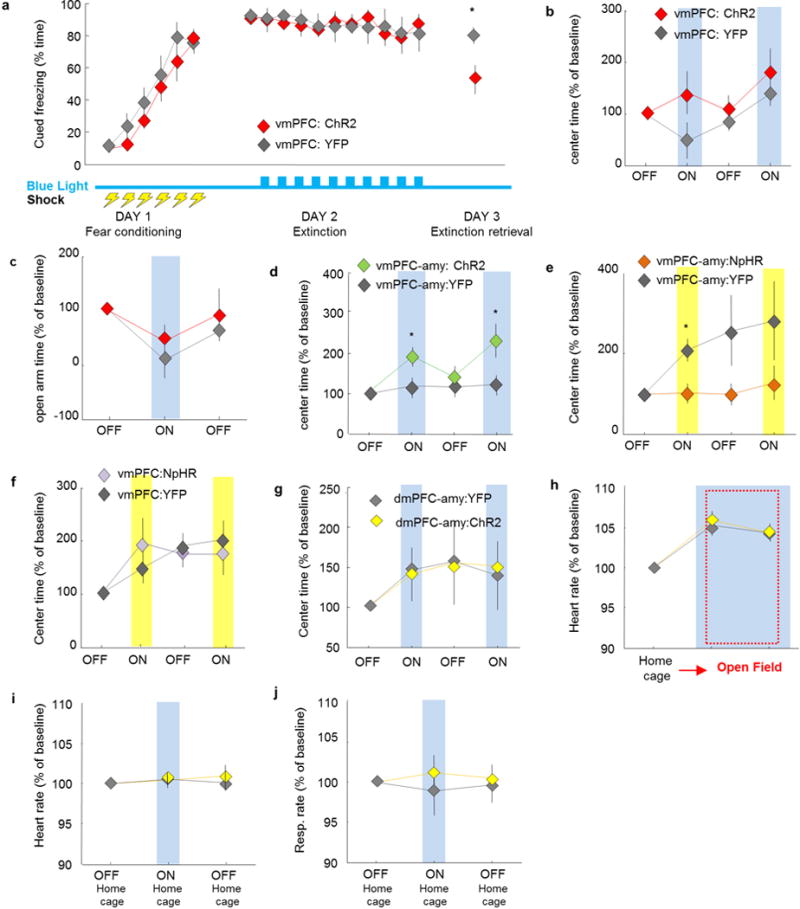
Optogenetic manipulations involving the mPFC and its projections to the amygdala in fear and anxiety paradigms **a**, Mice were fear conditioned to six tone-shock (0.7 mA, 2-s shocks) pairings on day 1 (fear acquisition). On day 2 (fear extinction) animals were exposed to the tone in a different context for 11 trials. Blue light was delivered only on day 2, for trials 2–11. vmPFC:ChR2 mice froze less than control mice during day 3 (extinction retrieval). **b**, **c**, Blue light delivery did not change avoidance of open spaces in vmPFC:ChR2 mice relative to control mice in the open field (**b**) or the EPM (**c**). *n* = 7 vmPFC:ChR2 and 7 vmPFC:YFP mice (**a–c**). **d**, Excitation of the vmPFC–amygdala projection with blue light increased exploration of the centre of the open field in vmPFC–amygdala:ChR2 mice relative to controls. Two-way repeated measures ANOVA, opsin × epoch interaction, *F*_3,68_ = 3.1, *P* = 0.03, post hoc Wilcoxon rank sum test *P* = 0.002. *n* = 12 vmPFC–amygdala:ChR2 and 13 vmPFC–amygdala:YFP mice. **e**, Inhibition of the same projection in vmPFC–amygdala:NpHR mice with yellow light increased avoidance of open spaces. Two-way repeated-measures ANOVA, main effect of opsin, *F*_3,68_ = 7.26, *P =* 0.008, post-hoc Wilcoxon rank sum test *P =* 0.03. *n* = 14 vmPFC–amygdala:NpHR and 11 vmPFC–amygdalal:YFP mice. **f**, Same as **e**, but for inhibition of vmPFC cell bodies. *n* = 11 vmPFC:NpHR and 8 vmPFC:YFP mice. **g–j**, Optogenetic stimulation of the dmPFC–amygdala projection did not alter behaviour in the open field (**g**), heart rate in the open field (**h**), heart rate in the home cage (**i**), or respiration rates in the home cage (**j**). Blue light stimulation epochs are labelled ON and/or with a blue bar. *n = 7* dmPFC–amygdala:YFP and 7 mPFC–amygdala:ChR2 mice. **b–j**, Data are plotted in 5-min consecutive intervals. Light delivery epochs are labelled ON and/or with a blue or yellow bar. *P < 0.05, Wilcoxon rank sum test; error bars, ± s.e.m.; *n* refers to biological replicates.

**Figure 1:**
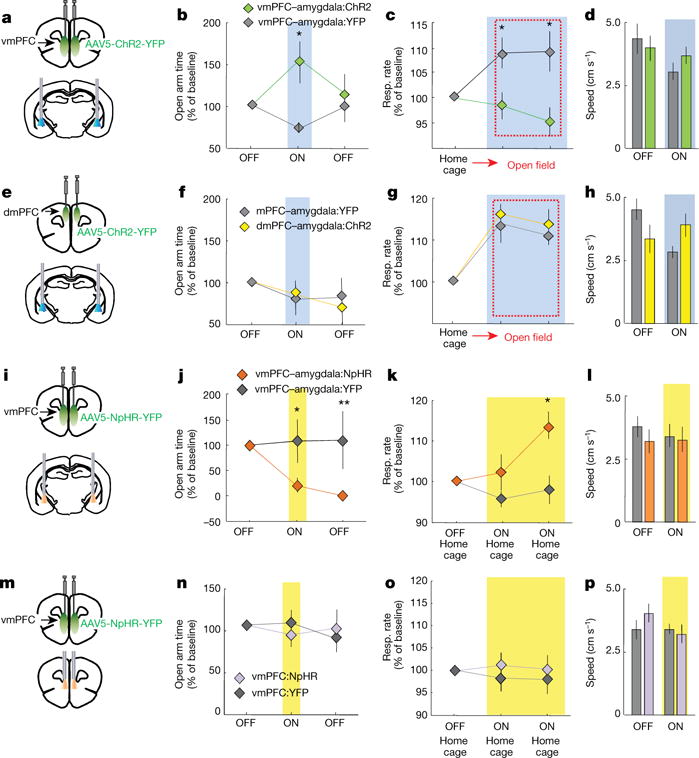
Activating vmPFC but not dmPFC terminals in amygdala decreases anxiety **a**, vmPFC-amy:ChR2 mice expressing ChR2 in vmPFC with fibreoptics above amygdala. **b**, Blue light increased exploration of open arms in vmPFC-amy:ChR2 mice. *n* = 11 v mPFC–amygdala:C hR2; 10 vmPFC–amygdala:YFP mice. **c**, Respiratory rates recorded for 5 min in home cage and 10 min in open field (red dashed rectangle). Blue light in vmPFC–amygdala:ChR2 mice prevented increases in respiratory rate in open field (OFT) relative to home cage, without altering locomotion (**d**). *n* = 11 vmPFC–amygdala:ChR2; 6 vmPFC–amygdala:YFP mice. **e**–**h**, Same as **a**–**d**, but for dmPFC–amygdala projections. *n* = 7 dmPFC–amygdala:YFP; 7 mPFC–amygdala:ChR2 mice. **i**, vmPFC–amygdala:NpHR mice expressing eNpHR3.0 (abbreviated in all figures as NpHR) in vmPFC with fibre optics above amygdala. **j**, **k**, Yellow light decreased exploration of open arms (**j**; *n* = 8 vmPFC–amygdala:NpHR; 7 vmPFC–amygdala:YFP) and increased home cage respiratory rates (**k**; *n =* 6 vmPFC-amygdala:NpHR; 6 vmPFC–amygdala:YFP). **l**, Yellow light did not alter overall locomotion *(n* = 14 vmPFC-amygdala:NpHR; 11 vmPFC–amygdala:YFP). **m**, vmPFC:NpHR mice with fibre optics above vmPFC. **n**–**p**, Yellow light did not change exploration of EPM (**n**), home cage respiratory rates (**o**) or speed (**p**). *n* = 11 vmPFC:NpHR; 8 vmPFC:YFP mice. *P < 0.05; **P < 0.01, Two-sided Wilcoxon test. Data plotted in 5-min consecutive intervals. Light delivery epochs labelled ON or with blue/yellow bars. Error bars, mean ± s.e.m.

Increased anxiety involves alterations in physiology and behaviour (for example, increases in respiratory rate and risk-avoidance)^24^. In rodents, avoidance of open arms of the elevated plus maze (EPM) is a measure of anxiety-related behaviour^24,25^. We expressed channelrhodopsin ChR2-H134R in mouse vmPFC (Extended Data Fig. 1a, b, Supplementary Note 1) and implanted fibre-optic cannulae above the amygdala (vmPFC–amygdala:ChR2 mice, Fig. 1a) or above vmPFC (vmPFC:ChR2 mice). vmPFC–amygdala activation decreased avoidance of open spaces in EPM (two-way repeated measures analysis of variance (ANOVA), opsin × epoch interaction, *F*_2,37_ = 3.1, *P* = 0.04, post-hoc Wilcoxon rank-sum test *P* = 0.009; Fig. 1b, see Extended Data Table 1 for absolute values) and open field (Extended Data Fig. 2d). Projection-targeting appeared to resolve a distinct cell population, since directly exciting the entire vmPFC was not anxiolytic (Extended Data Fig. 2b, c).

**Extended Data Figure 3:**
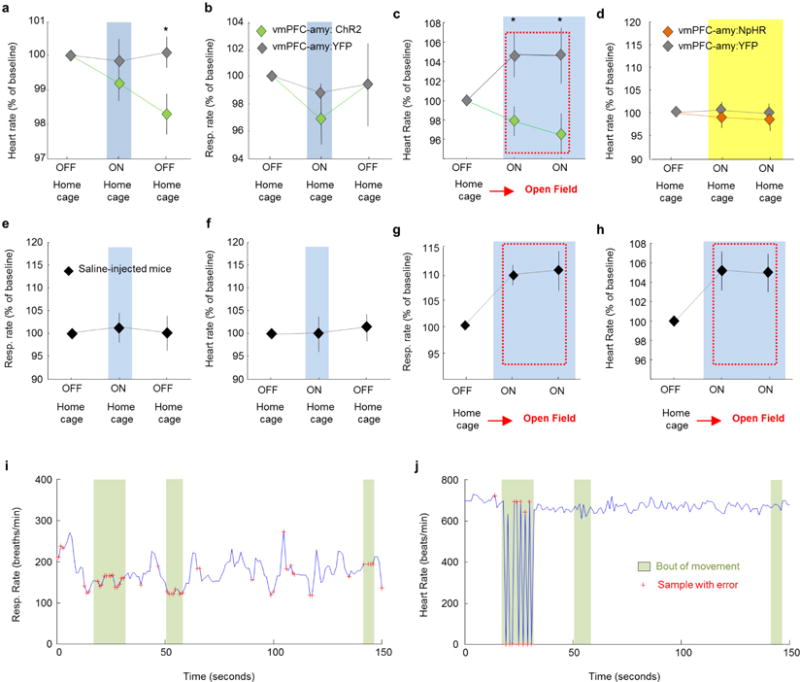
Heart and respiratory rate changes elicited by optogenetic manipulation of vmPFC fibres in the amygdala **a**, **b**, Optogenetic stimulation of vmPFC fibres in the amygdala in the home cage did not significantly alter heart (**a**) or respiratory rate (**b**) in mice in the home cage during the light ON period. Nevertheless, a downward trend was observed for both measurements during delivery of blue light. *n* = 12 vmPFC–amygdala:ChR2 and 6 vmPFC–amygdala:YFP mice. **c**, Blue light delivery in vmPFC–amygdala:ChR2, but not control, mice prevented increases in heart rate in the open field test (OFT) relative to the home cage. Two-way repeated measures ANOVA, main effect of opsin, *F*_2,29_ = 10.98, *P* = 0.0019, post-hoc Wilcoxon rank sum test *P* = 0.04; *n* = 11 vmPFC–amygdala:ChR2 and 6 vmPFC–amygdala:YFP mice. **d**, Inhibition of the vmPFC–amygdala projection in vmPFC–amygdala:NpHR mice with yellow light in the home cage did not alter heart rate. *n* = 6 vmPFC–amygdala:NpHR; 6 vmPFC–amygdala:YFP mice. **e–h**, Mice were injected with saline in the vmPFC. Fibre optics were placed above the BMA. **e**, **f**, Delivery of blue light did not alter respiratory rate (**e**) or heart rate (**f**) in the home cage. **g**, **h**, Respiratory rate (**g**) and heart rate (**h**) increased, relative to the home cage, when mice were placed in the anxiogenic open field. Blue light delivery did not prevent the increase in respiratory and heart rate observed in the open field. *n* = 7 sham mice. **a–h**, Data are plotted in 5-min consecutive intervals. Light stimulation epochs are labelled with ON and with a blue or yellow bar. **i**, **j**, Example raw traces of respiratory (**i**) and heart rate (**j**) recorded at 1 Hz obtained from a freely moving mouse through pulse oximetry. Movement bouts are shown in green, and single samples with errors due to motion artefacts are shown as red crosses. Error samples are detected automatically by software (Starr Life Sciences). **i**, Most error samples occur during movement bouts and a few errors can be seen outside of movement bouts in the respiratory rate trace. **j**, Heart rate recordings are generally stable and errors occur only during prolonged and large movement bouts. Samples with errors were not used in any other plot or data analysis. Representative traces from one mouse. Error bars, ± s.e.m.; *n* refers to biological replicates.

**Extended Data Figure 4:**
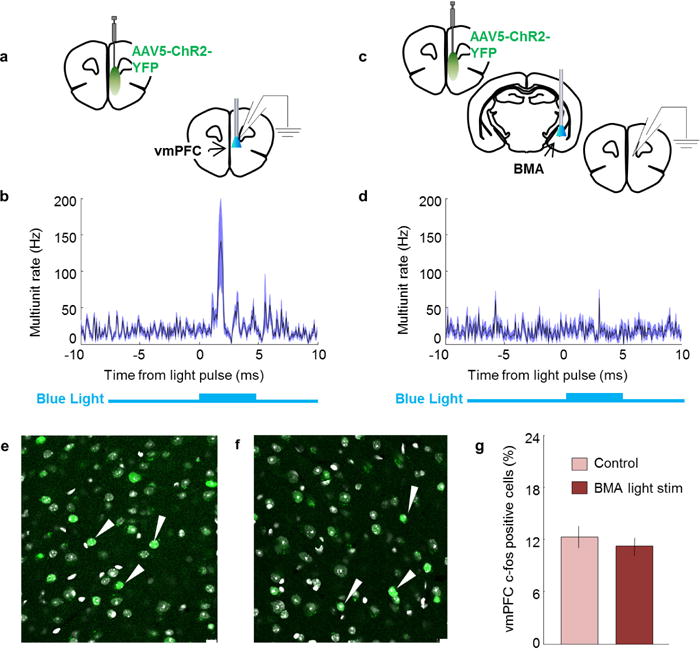
Stimulation of ChR2-expressing vmPFC terminals in the basomedial amygdala: lack of detection of antidromic spikes in vmPFC **a**, Mice were injected with AAV5-CamK2α -ChR2-YFP in the vmPFC. Blue light was delivered above the vmPFC. Simultaneous *in vivo* anaesthetized recordings under isoflurane were obtained from the vmPFC. **b**, Average of 64 recording sites in the mPFC showing that blue light elicited orthodromic spikes in ChR2-expressing cortical cells. **c**, Same as **a**, but blue light was delivered to ChR2-expressing vmPFC terminals in the BMA while recordings were obtained from the mPFC. **d**, Average of 64 recording mPFC sites showing that multiunit activity in the mPFC did not detectably increase following excitation of vmPFC terminals in the amygdala. Recordings with delivery of blue light to the vmPFC (**a**) or BMA (**c**) were obtained from the same mice. The 5 ms blue light pulse is shown in blue below the graph. A 32-site recording electrode probe was used to target deep cortical layers. *n* = 64 sites from 2 animals (**b**, **d**). **e–f**, Compared to baseline controls (**e**), stimulation of ChR2-expressing vmPFC fibres in the BMA of freely behaving awake animals (**f**) did not change c-Fos expression in deep layers of the vmPFC (layers 5 and 6). **g**, Summary bar graph showing the mean percentage of c-Fos positive cells in control animals and mice with stimulation of vmPFC fibres in the BMA. *n* = 5 animals for each group. **e–f**, Arrowheads indicate examples of c-Fos-expressing cells. Scale bar, 10 μm; error bars, ± s.e.m.; *n* refers to biological replicates.

Respiratory rates increased during exploration of the anxiogenic open field (relative to home cage)^24,26,27^ in control animals (second and third epochs in Fig. 1c, g marked by a dashed red line, Fig. 1c; see Extended Data Fig. 3 for heart rate (a), sham-injected animals (e–h) and raw traces (i, j)). vmPFC–amygdala activation suppressed this increase in respiratory rate (Fig. 1c; two-way repeated measures ANOVA, main effect of opsin, *F*_2,29_ = 11.7, *P* = 0.0014, post-hoc Wilcoxon rank-sum test, *P* = 0.03) without changing locomotion (Fig. 1d), or respiratory rate in the home cage (Extended Data Fig. 3b), suggesting suppression of respiratory rate only in anxiogenic settings (Supplementary Note 2). Antidromic spikes in PFC were not readily detected following optogenetic excitation of vmPFC–amygdala: ChR2 terminals (Extended Data Fig. 4). Dorsal mPFC (dmPFC) was investigated next. dmPFC–amygdala: YFP mice did not exhibit labelled fibres in hypothalamus, in contrast to vmPFC–amygdala: YFP mice (Extended Data Fig. 1c, g), supporting correct anatomical targeting^9^ (Supplementary Note 1). Upon optical stimulation, dmPFC–amygdala: ChR2 mice showed no changes in behavioural (Fig. 1f, h and Extended Data Fig. 2g) or physiological (Fig. 1g and Extended Data Fig. 2h–j) measures of anxiety; consistent with prior reports^28,29^.

To evaluate the necessity of endogenous vmPFC–amygdala activity for anxiolysis, we expressed the inhibitory halorhodopsin eNpHR3.0^12,24^ in vmPFC (Fig. 1i). vmPFC–amygdala inhibition increased avoidance of open spaces in vmPFC–amygdala:eNpHR3.0 mice in EPM (Fig. 1j; two-way repeated measures ANOVA, main effect of opsin × epoch interaction, *F*_2,25_ = 5.0, *P* = 0.014, post hoc Wilcoxon rank-sum test *P* = 0.02) and open field (Extended Data Fig. 2e). Moreover, vmPFC–amygdala inhibition in the home cage increased respiratory rate (Fig. 1k), but not locomotion (Fig. 1l) or heart rate (Extended Data Fig. 3d). Conversely, nonspecific mPFC inhibition did not change avoidance of open spaces (Fig. 1m, n and Extended Data Fig. 2f) or respiratory rate (Fig. 1o), revealing that vmPFC–amygdala endogenous activity regulates anxiety states.

## vmPFC–amygdala promotes fear extinction

**Figure 2:**
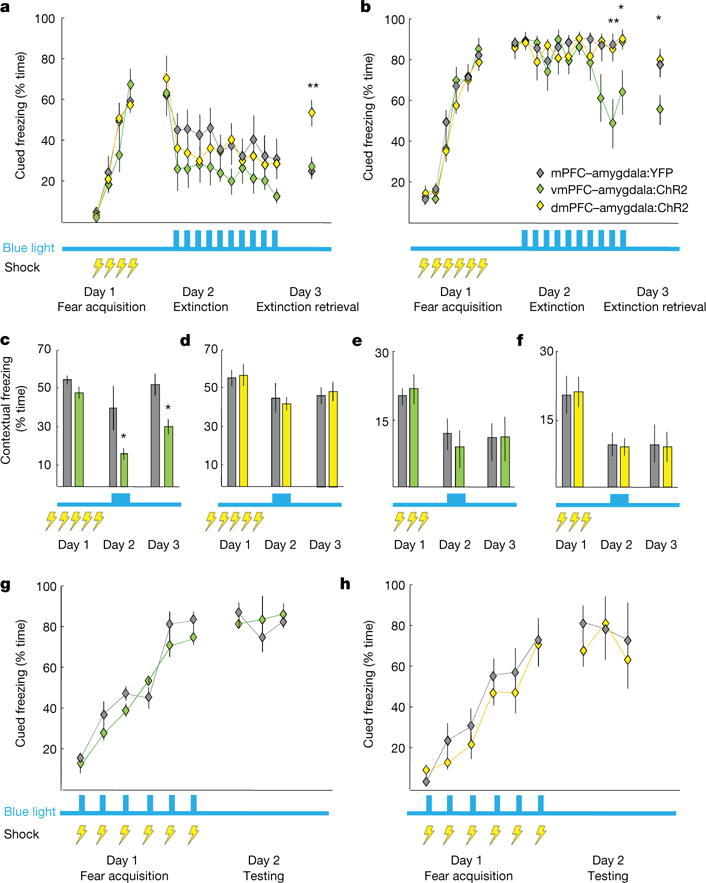
Activation of vmPFC–amygdala and dmPFC–amygdala projections: opposite effects on cued fear **a**, Mice fear conditioned to four tone-shock pairings (0.4 mA/1-s shocks) on day 1; extinction on day 2. Blue light delivered on day 2; trials 2–11. dmPFC–amygdala:ChR2 mice froze more than controls during day 3. *n* = 11 mPFC–amygdala:YFP, 7 vmPFC–amygdala:ChR2, 13 dmPFC–amygdala:ChR2 mice. **b**, Same as **a**, but six tone-shock pairings (0.7 mA/2-s shocks). vmPFC–amygdala:ChR2 mice froze less than controls at end of extinction and during extinction retrieval. *n* = 12 mPFC–amygdala:YFP, 9 vmPFC–amygdala:ChR2, 10 dmPFC–amygdala:ChR2 mice. **c**, Mice received five foot shocks (0.4 mA/1-s shocks) during contextual fear acquisition. Light decreased freezing in vmPFC–amygdala:ChR2 mice. *n* = 8 vmPFC–amygdala:YFP; 9 vmPFC–amygdala:ChR2 mice. **d**, Same as **c**, but dmPFC–amygdala mice *(n* = 8 dmPFC-amygdala:YFP; 8 dmPFC–amygdala:ChR2 mice). **e**, **f**, Contextual fear conditioning: three shocks, 0.4 mA/1 s. Delivery of light during day 2 did not alter freezing in vmPFC–amygdala:ChR2 (**e**) or dmPFC–amygdala:ChR2 (**f**) mice. ***e****, n = 7* vmPFC–amygdala:ChR2; 6 vmPFC–amygdala:YFP mice. **f**, *n = 7* dmPFC–amygdala:ChR2; 7 dmPFC–amygdala:YFP mice. **g**, **h**, Delivery of light during fear acquisition (0.7 mA/2-s shocks) did not alter freezing in vmPFC–amygdala:ChR2 (**g**) or dmPFC–amygdala:ChR2 (**h**) mice. **g**, **h**, *n =* 8, all cohorts; *P < 0.05, #*P* < 0.01; two-sided Wilcoxon test. Error bars, mean ± s.e.m.

To investigate if this top-down projection controlled fear-conditioning, we employed mild cued fear-conditioning to avoid ceiling effects on freezing. vmPFC–amygdala activation during extinction increased freezing in dmPFC–amygdala:ChR2 mice during extinction retrieval (Fig. 2a, P = 0.01, Wilcoxon rank-sum test). We next employed stronger cued fear-conditioning to eliminate floor effects, allowing detection of freezing suppression. vmPFC–amygdala activation enhanced cued fear extinction (two-way repeated-measures ANOVA, *F*_18, 250_ = 10.73, *P* = 0.0012 for group type) and extinction retrieval (*P* = 0.04, Wilcoxon rank sum test; Fig. 2b); increased extinction retrieval was also seen following stimulation of vmPFC somata (Extended Data Fig. 2a)^7^. vmPFC–amygdala activation also decreased freezing in contextual fear extinction and extinction retrieval (Fig. 2c, two-way repeated-measures ANOVA; significant effect of group type, *F*_2,29_ = 8.7, *P* = 0.009). In contrast, dmPFC–amygdala activation did not alter contextual freezing (Fig. 2d, f).

**Extended Data Figure 5:**
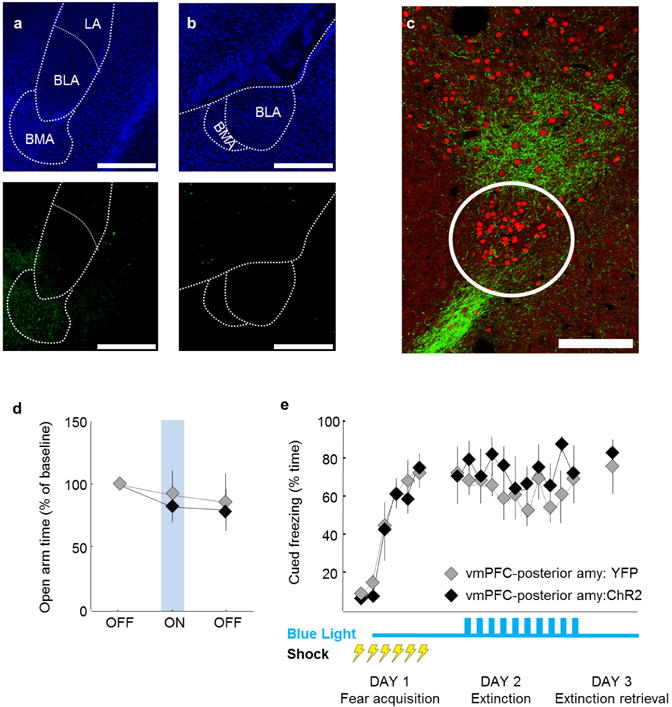
vmPFC innervation of the amygdala in mice and rats **a**, **b**, Mice were injected with AAV5-CamK2α -YFP in the vmPFC and fibres were imaged in the amygdala. **a**, Top, DAPI-stained amygdala section. Bottom, vmPFC fibres in the BMA 1.3 mm posterior from bregma. This coordinate was used for the fibre optic implantation in the vmPFC–amygdala behavioural cohorts. **b**, Same as **a**, but for a more posterior section (2.3 mm from bregma), showing no prominent vmPFC fibres of passage that traverse the BMA and terminate elsewhere. Nuclei were stained with DAPI, *n* = 4 mice. Scale bar, 0.5 mm. **c**, Rats were injected with AAV5-CaMK2α -SSFO-YFP in the vmPFC (infralimbic cortex). Six months following viral injection brain slices were stained for FoxP2 to identify ITCs (red). The representative image shows vmPFC fibres (green) surrounding an ITC cluster (circled in white). Note that the vmPFC does not strongly innervate the ITCs in rats. Nevertheless, a sparse vmPFC–ITC projection can be seen. Image from one representative animal chosen from *n* = 3 rats. Scale bar, 100 μm. **d**, **e**, Mice were injected with AAV5-CamK2α -ChR2-YFP in the vmPFC. Fibre optics were placed above the amygdala (amy), but 500 μm posterior to the implants shown in Fig. 1. Delivery of blue light to this posterior amygdala site did not alter exploration of the open arms in the elevated plus maze (**d**) or freezing in cued fear conditioning (**e**), suggesting that activation of vmPFC fibres of passage that go beyond the amygdala do not have an important role in regulating anxiety and fear. *n* = 7 vmPFC–posterior amygdala:YFP and 8 vmPFC–posterior amygdala:ChR2 mice. Error bars, ± s.e.m.; *n* refers to biological replicates.

Behavioural effects (Figs 1 and 2) were not attributable to activation of fibres-of-passage through amygdala, as vmPFC fibres were not observed in posterior sections of amygdala, revealing instead more proximal termination (Extended Data Fig. 5a, b); moreover, photo-stimulation even 500 μ m posterior to the coordinates used in the above experiments did not modulate anxiety or freezing (Extended Data Fig. 5d, e). These data reveal the vmPFC–amygdala projection suppresses fear-related freezing and high-anxiety states, while the dmPFC–amygdala projection selectively antagonizes only cued fear-extinction. Activation of either dmPFC or vmPFC projections to amygdala during cued fear acquisition did not alter freezing (Fig. 2g, h), in agreement with reports showing lack of mPFC involvement in fear acquisition^30,31^.

## BMA mediates vmPFC top-down control

**Extended Data Figure 6:**
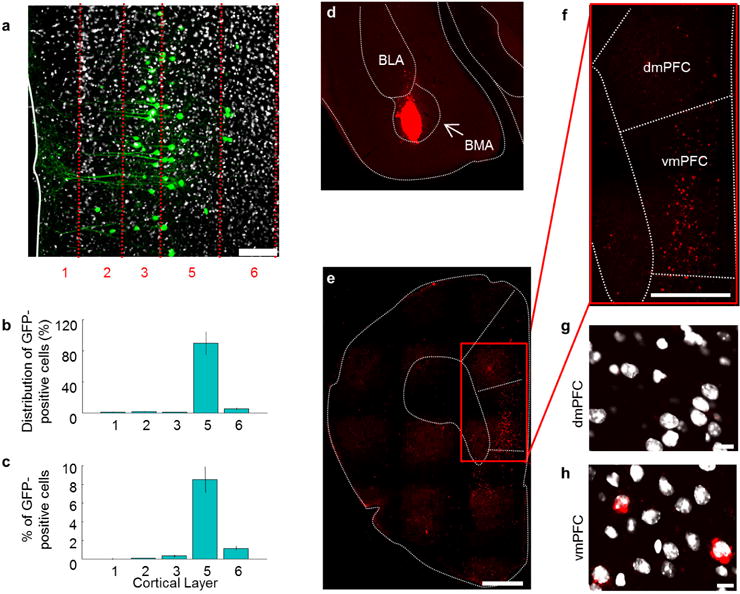
Quantification of BMA-projecting vmPFC neurons **a–c**, Mice were injected with the retrogradely propagating ΔG rabies-GFP virus in the basomedial amygdala (BMA). **a**, Ten days after viral infusion, retrogradely labelled vmPFC cells can be seen expressing GFP. The number of GFP-expressing vmPFC cells was quantified across layers, both as a percentage of all GFP-positive cells (**b**) and as a percentage of all vmPFC cells (**c**) (counting labelled and unlab elled cells). *n* = 4 mice; scale bar, 75 μm (**a**). **d**, Mice were injected with retrobeads in the BMA. **e**, Image of a coronal section containing the mPFC. Note the presence of retrobead-containing cells in the vmPFC. **f**, Expanded image of the zone demarcated by a red rectangle in **e**. Labelled cells can be seen in the vmPFC, but not the dmPFC. **g**, **h**, Confocal image showing unlabelled cells in the dmPFC (**g**) and labelled cells in the vmPFC (**h**). **a**, **g**, **h**, Nuclei were stained with DAPI. *n* = 5 mice (**d–h**). Scale bars, 250 μm (**d**); 500 μm (**e**, **f**); 10 μm (**g**, **h**). Error bars, ± s.e.m.; *n* refers to biological replicates.

**Figure 3:**
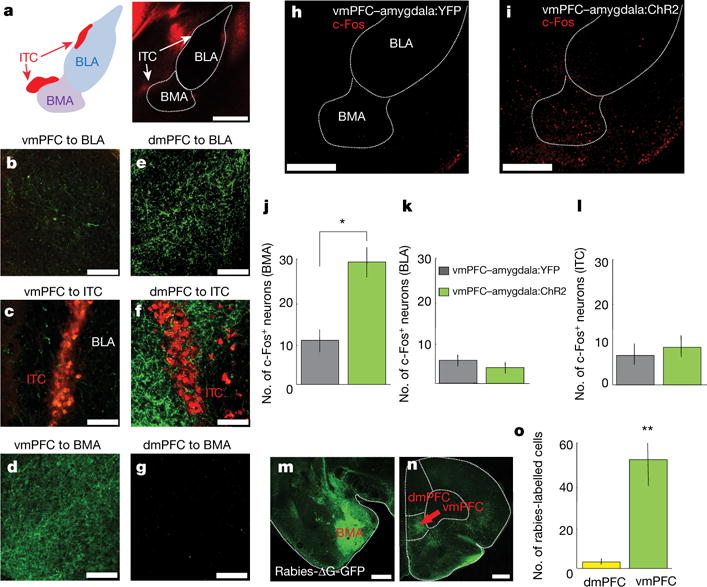
Basomedial amygdala: major target of vmPFC in amygdala **a**, Left: location of amygdala nuclei. Right: *lox*-tdTomato reporter line crossed with EY266 D1R∷Cre line for ITC visualization. Scale bar, 500 μm. **b–d**, AAV5-CamK2α∷ChR2-YFP injected in vmPFC; vmPFC fibres imaged in BLA (**b**), ITC (**c**) and BMA (**d**). **a–d**, Representative images from one mouse chosen out of *n* = 7 mice. **e–g**, Same as **b–d**, but for dmPFC. **e–g**, Representative images from one mouse chosen out of *n* = 7 mice. Scale bars, 50 μm (**c**, **f**); 100 μm (**b**, **e**, **d**, **g**). **h**–**l**, c-Fos-expressing cells (red) counted in BMA (**j**), BLA (**k**) and ITCs (**l**) following vmPFC–amygdala activation; increased c-Fos can be seen in BMA of vmPFC–amygdala:ChR2 mice. *n =* 5 mice, for each group, 4 slices per mouse. **m–o**, Rabies-ΔG-GFP injected in BMA (**m**); retrogradely labelled cells counted (**n–o**). *n =* 4 mice (**o**). Scale bar, 500 μm (**h**, **i**, **m**, **n**). *P < 0.05, **P < 0.01, two-sided Wilcoxon test. Error bars, mean ± s.e.m.

**Figure 4:**
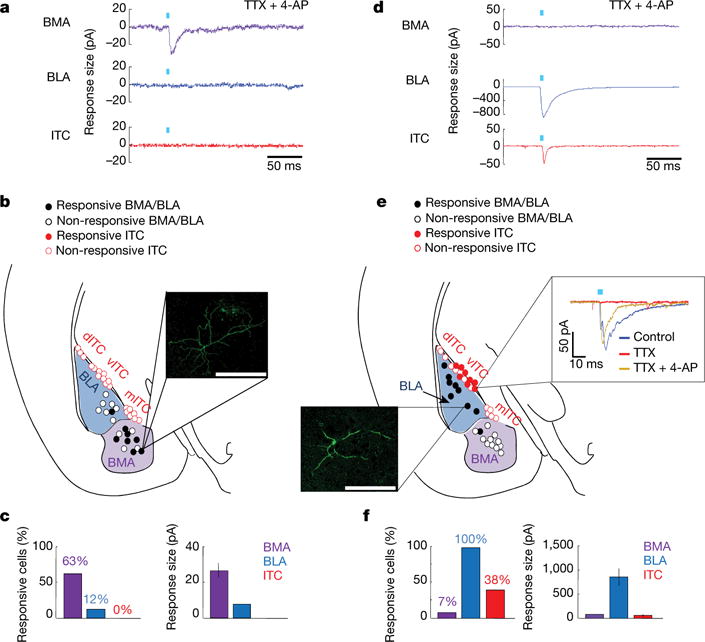
Functional connectivity: mPFC inputs to amygdala **a**, Example traces from one mouse showing stimulation of ChR2-expressing vmPFC terminals in amygdala (acute slice) elicited responses in BMA, but not BLA or ITCs. Recordings in TTX and 4-AP to abolish polysynaptic responses. **b**, Locations of recorded cells. Inset: responsive biocytin-filled BMA cell. dITC, vITC and mITC: dorsal, ventral and main ITC clusters. *n* = 11 BMA, 8 BLA and 19 ITC cells. **c**, Mean percentage of responsive cells and response sizes. *n* = 4 mice (**a**–**c**). **d**–**f**, Same as **a**–**c**, but dmPFC terminal stimulation. *n* = 4 mice (**d**–**f**). **b**, **e**, Inset scale bar, 250 μm. Error bars, mean ± s.e.m.

We next sought precise circuit targets underlying this top-down modulation. We observed sparse vmPFC fibres in BLA and ITCs (Fig. 3b, c; CLARITY data, Supplementary Video 1; Extended Data Fig. 5c), but prominent investment of basomedial amygdala (BMA; Fig. 3d)^9,32,33^. Conversely, dmPFC fibres strongly invested BLA (and ITCs, not previously reported), while sparing the BMA (Fig. 3e–g). Accordingly, vmPFC–amygdala activation induced c-Fos in BMA, but not in BLA or ITCs (Fig. 3h–l). We next injected the retrogradely propagating virus rabies-ΔG-GFP into BMA and identified a strong input specifically from vmPFC (Fig. 3m–o), predominantly from layer V (Extended Data Fig. 6a–c); similar results were obtained with retrobeads (Extended Data Fig. 6d–h). Finally, we obtained whole-cell recordings from amygdala cells during optical stimulation of ChR2-expressing mPFC terminals (in tetrodotoxin and 4-aminopyridine to abolish polysynaptic responses); stimulation of vmPFC terminals elicited monosynaptic responses in BMA (7/11 cells), in contrast to BLA (1/8 cells) or ITCs (0/19 cells) (Fig. 4a–c). Conversely, dmPFC terminals spared BMA but elicited monosynaptic responses in BLA and the ventral ITC cluster (Fig. 4d–f).

**Extended Data Figure 7:**
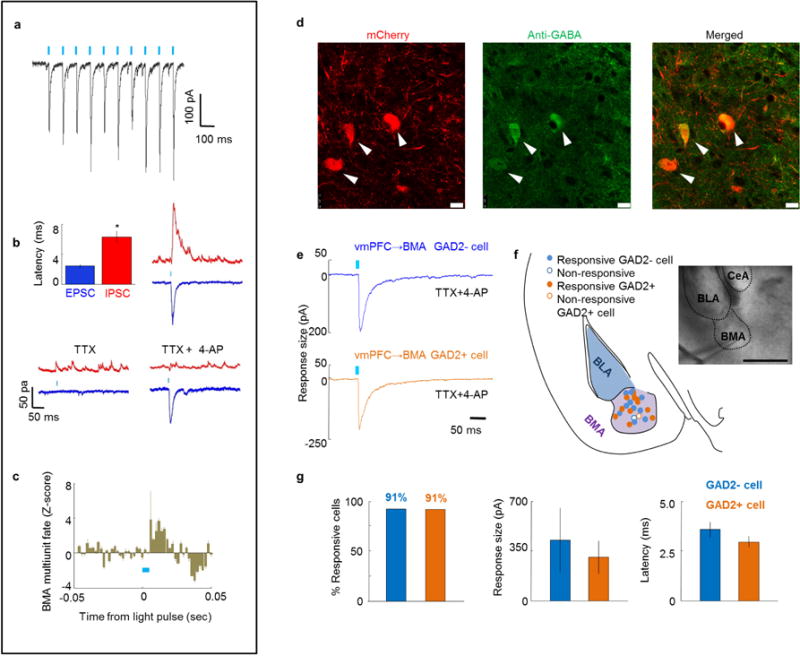
Characterization of the vmPFC–BMA projection by optical stimulation of vmPFC terminals *in vivo* and *in vitro* **a**, Example trace from one mouse showing responses in a BMA cell following a train of 5-ms 10 Hz pulses in an acute brain slice with ChR2-expressing vmPFC terminals. These were the same parameters used for behavioural optogenetic experiments. **b**, Optogenetic stimulation of vmPFC fibres in the BMA in acute brain slices elicited both IPSCs (red) and EPSCs (blue), which had significantly different latencies. TTX abolished both IPSCs and EPSCs. 4-AP was added in the presence of TTX to rescue monosynaptic responses. Note that 4-AP rescued the EPSC, but not the IPSC. *n* = 7 cells from *n* = 2 mice. **c**, BMA multiunit recordings were obtained in awake behaving mice during optical stimulation of ChR2-expressing vmPFC terminals. Activation of vmPFC terminals dramatically increased firing rates in the BMA. The graph shown is an average of *n* = 14 multiunit recordings from *n* = 4 mice. **d**, A GAD2-Cre mouse was injected with AAV5-DIO-mCherry in the BMA. First panel shows antibody staining against GABA. Middle panel shows expression of mCherry in Cre-expressing cells. Last panel shows a merged photo of the first two panels. Note overlap of mCherry expression and GABA staining. Arrowheads show examples of double-labelled cells. *n =* 5 mice. **e**, Example traces from one mouse (chosen from *n =* 3 mice) showing stimulation of ChR2-expressing vmPFC terminals in amygdala acute slices elicits responses in both GAD2 negative (putative excitatory projection cells) and positive cells (inhibitory interneurons). Recordings were done in the presence of TTX and 4-AP to abolish polysynaptic responses. **f**, Scheme displaying the location of all recorded cells. Responsive cells are shown as filled circles. Inset shows a BMA cell being patched. Inset scale bar, 10 μm. **g**, Left: mean percentage of responsive cells. Middle: average response size of recorded cells. Right: Average latency of recorded responses relative to the start of the light pulse., *n =* 12 GAD2 positive and 12 GAD2 negative cells (from *n =* 3 mice) (**e–g**). 4-AP, 4-aminopyridine; TTX, tetrodotoxin; IPSC, inhibitory postsynaptic current; EPSC, excitatory postsynaptic current. **a**, **b**, **c**, **e**, A 5 ms pulse of blue light (indicated by a blue tick mark) was used to elicit stimulation. *P < 0.05; error bars, ± s.e.m.; *n* refers to biological replicates.

**Extended Data Figure 8:**
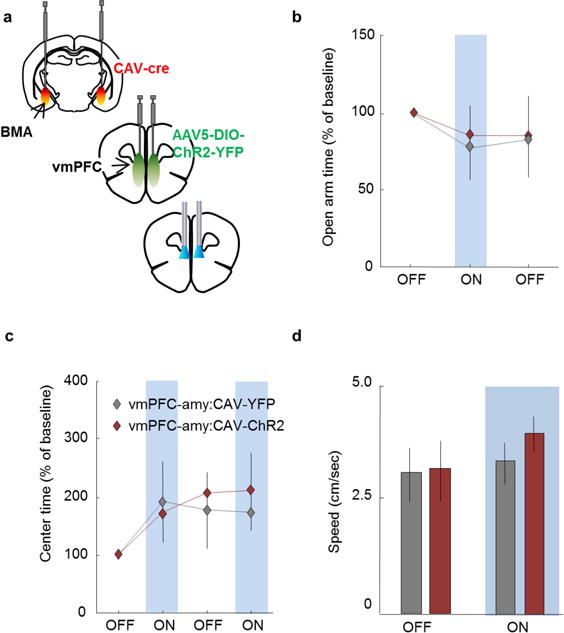
Activation of BMA-projecting vmPFC cells decreases cued fear **a**, Mice were injected with the retrogradely propagating canine adenovirus encoding Cre recombinase (CAV-Cre) in the BMA. Mice were also injected with a viral vector that induces expression of ChR2-YFP or YFP only in the presence of Cre recombinase (AAV5-DIO-ChR2-YFP). Mice were implanted bilaterally with fibre optics above the vmPFC for delivery of blue light. **b–d**, Delivery of blue light (10 Hz, 5-ms pulses at 10 mW) did not alter exploration of the open arms in the EPM (**b**), the centre of the open field (c), or speed (**d**). *n* = 7 vmPFC–amygdala:CAV-YFP and 8 vmPFC–amygdala:CAV-ChR2 mice. Error bars, ± s.e.m.; *n* refers to biological replicates.

**Figure 5:**
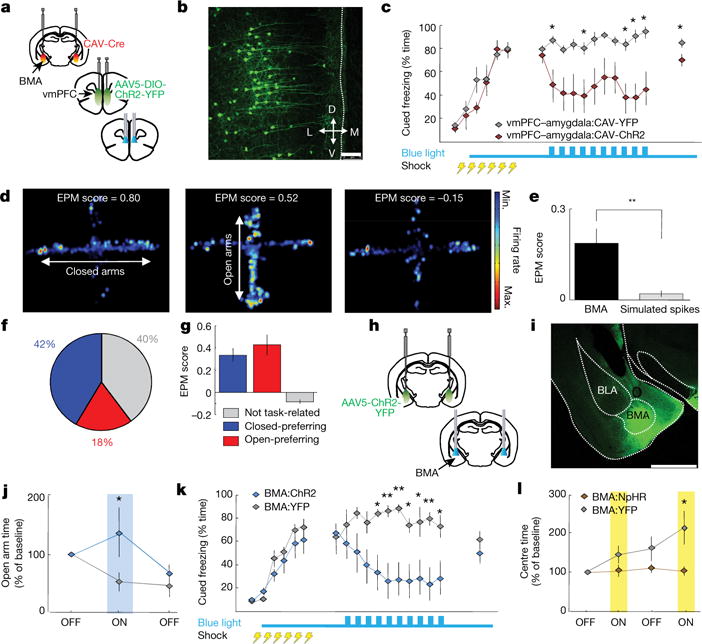
BMA cells encode anxiety-related contextual features, decrease anxiety and decrease freezing **a**, CAV-Cre in BMA and AAV5-DIO-ChR2 in vmPFC. **b**, YFP-expression in BMA-projecting vmPFC neurons. Scale bar, 75 μm. **c**, Light-decreased freezing. **b**, **c**, *n* = 7 vmPFC–amygdala:CAV-YFP, 8 vmPFC–amygdala:CAV-ChR2. **d**, Heat maps of representative cells preferentially firing in closed arms (left) or open arms (middle), or with no arm-type preference (right). **e**, EPM scores (strong encoding of arm-type) for BMA units versus simulated spike trains; *n* = 3 single-unit examples chosen from 38 BMA units recorded from *n* = 4 mice. **f**, Distribution of open- and closed-arm-preferring cells (EPM score > 0) versus no task-related firing (EPM score ≤ 0). **g**, Average EPM scores. **d–g**, *n =* 38 BMA single units from *n =* 4 mice. **h**, **i**, BMA:ChR2 mice expressing ChR2 in BMA with fibre optics above BMA. Scale bar, 1 mm. **j**, **k**, BMA activation increased open-arm exploration (**j)** and decreased freezing (**k**). **i**, **j**, *n =* 8 BMA:ChR2; 9 BMA:YFP. **k**, *n =* 6 BMA:ChR2; 7 BMA:YFP. **l**, Delivery of light increased OFT centre-avoidance in BMA:NpHR mice. *n =* 8 BMA:NpHR; 7 BMA:YFP; *P < 0.05, **P < 0.01, two-sided Wilcoxon test. Error bars, mean ± s.e.m.

We next found that BMA cells *in vitro* could follow 10 Hz stimulation of vmPFC fibres (Extended Data Fig. 7a), and that vmPFC–amygdala activation elicited initial BMA excitation followed by inhibition *in vitro* (Extended Data Fig. 7b) and *in vivo* (Extended Data Fig. 7c). The inhibition was probably mediated by directly recruited GAD2-expressing cells in BMA, since 91% of these receive vmPFC inputs (Extended Data Fig. 7g). Finally, we tested if specific activation of vmPFC cell bodies that project to BMA suppressed freezing. We targeted BMA-projecting vmPFC cells by injecting the retrogradely propagating canine adenovirus (CAV) encoding Cre-recombinase into BMA (Extended Data Fig. 8, Supplementary Note 3) and directing AAV-DIO-ChR2-YFP as well as optical fibres into mPFC (Fig. 5a, b). Activation of these cells decreased cued freezing during extinction and extinction retrieval (Fig. 5c), demonstrating that BMA can account for the behavioural effects seen following vmPFC–amygdala activation.

## BMA inhibits fear and anxiety

**Extended Data Figure 9:**
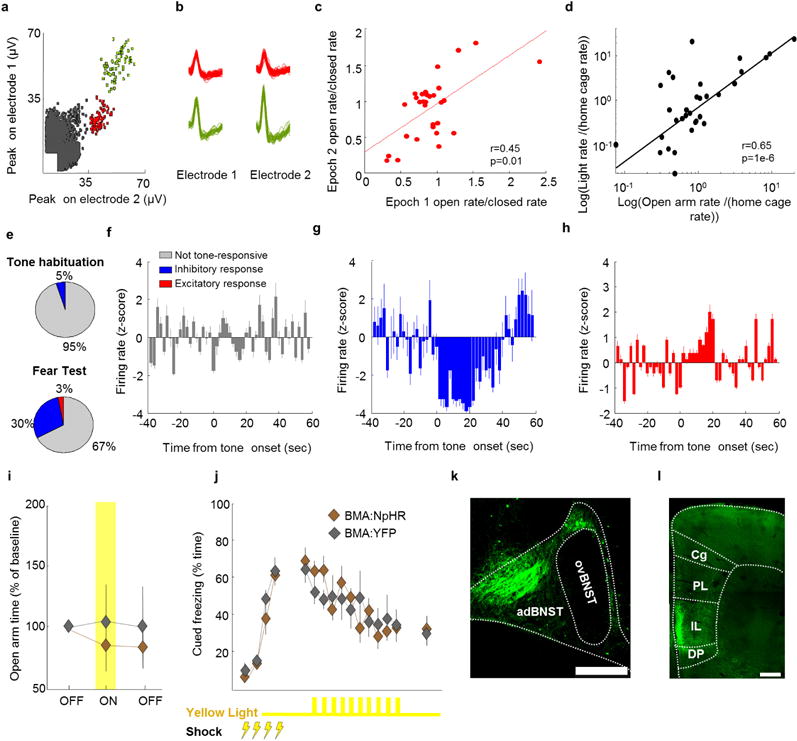
BMA activity and function in anxiety and fear paradigms **a**, Example isolated BMA single-unit spike clusters recorded with stereotrodes *in vivo*. **b**, Waveforms of the single-unit clusters shown in a, as recorded on each of the two electrodes comprising the stereotrode. **c**, Ratios of BMA neuron open arm/closed arm firing rates are shown for minutes 1 to 10 (epoch 1) and minutes 10 to 20 (epoch 2) of a 20 min exploration session of the elevated plus maze (EPM). Open/closed firing rate ratios are highly correlated across both epochs (*r* = 0.45, Spearman correlation), indicating that BMA firing patterns were stable throughout the entire 20 min session. **d**, The same cells shown in **c** were also recorded in the home cage and in the light/dark test. Firing rates in the light compartment of the light/dark test and the open arms of the EPM (plotted as fold-increase of rates from the non-anxiogenic home cage) were highly correlated (*r* = 0.65, Spearman correlation), indicating that BMA neurons respond similarly to anxiety induced by two different anxiogenic stimuli (bright lights and open areas). *n* = 38 cells from *n* = 4 mice (**a–d**). **e–h**, Recordings were obtained from basomedial amygdala (BMA) cells during presentation of a fear conditioned auditory tone. **e**, Top, distribution of responsive cells to the auditory tone before fear conditioning. Bottom, same as in upper panel, but for a fear recall test. The proportion of responsive cells increased following fear conditioning. Note that the vast majority of tone-responsive cells showed decreases in firing rate during the presentation of the fear-conditioned tone. **f**, Example cell that was not tone-responsive. **g**, **h**, Example cells that are inhibited **(g**) or excited (**h**) during tone presentation. **e**, *n* = 20 c**e**lls during habituation and 71 cells during fear recall. **f–h**, Data are an average of ten tone presentations for each of the three cells shown. *n* = 4 mice (**a–h**). **i**, Mice were injected with AAV5-CamK2α -NpHR-YFP in the BMA. **i**, **j**, Yellow light didn’t change behaviour in the elevated plus maze (**i**), or cued fear extinction (**j**). *n* = 8 BMA:NpHR and 7 BMA:YFP mice (**i**, **j**). **k**, Eight weeks after viral injections BMA projections can be seen in BMA:YFP mice in the anterodorsal bed nucleus of the stria terminalis (adBNST) but not in the oval BNST (ovBNST). **l**, Prominent BMA innervation was also visible in the infralimbic cortex (IL), but not in the prelimbic (PL), dorsal peduncular (DP) or cingulate cortices (Cg). Images from one representative mouse chosen from *n* = 9 BMA:YFP mice. Scale bars, 250 μm (**a**, **b**); 500 μm (**k**, **l**). Error bars, ± s.e.m.; *n* refers to biological replicates.

If the BMA is naturally used to suppress fear and anxiety, then BMA spiking should differentiate safe and aversive contexts. We recorded BMA cells (Extended Data Fig. 9a, b) and used EPM scores to quantify arm-type encoding (Methods and refs 24,34). BMA neurons exhibited stable firing patterns (Extended Data Fig. 9c) with higher EPM scores than would be expected by chance (Fig. 5d, e; *P* = 0.01, Wilcoxon rank-sum test). In light–dark tests (LDT), where anxiety is associated with the light compartment of the apparatus^35^; the same BMA cells showed similar changes in firing rate in EPM open arms and LDT light compartments (Extended Data Fig. 9d), indicating that BMA neurons encode anxiety-associated contextual features. Among cells with positive EPM scores, neurons that fired preferentially in closed arms were over-represented (*P* < 0.05, binomial test) relative to open-arm-preferring cells (Fig. 5f). Similarly, a larger fraction of BMA cells (*P* < 0.01, binomial test) displayed inhibition versus excitation to fear-conditioned tones (Extended Data Fig. 9e). Consistent with this abundance of low-spiking BMA cells in actively aversive/anxiogenic settings, and the hypothesized countervailing role for excitatory anxiolytic inputs to BMA, we found BMA activation (Fig. 5h, i) decreased avoidance of open arms (Fig. 5j; two-way repeated-measures ANOVA, main effect of opsin, *F*_2,29_ = 4.9, *P* = 0.03, post hoc Wilcoxon rank-sum test *P* = 0.04) and freezing in cued fear (Fig. 5k; two-way repeated-measures ANOVA, main effect of opsin, *F*_9,99_ = 12.3, *P* = 0.004). Conversely, inhibition of BMA with eNpHR3.0 was anxiogenic in the open field (*P* < 0.05, main effect of opsin, two-way repeated-measures ANOVA, *F*_2,25_ = 4.1, *P* < 0.01, Fig. 5l), perhaps concordant with a role for the BMA in fear^36,37^.

**Figure 6:**
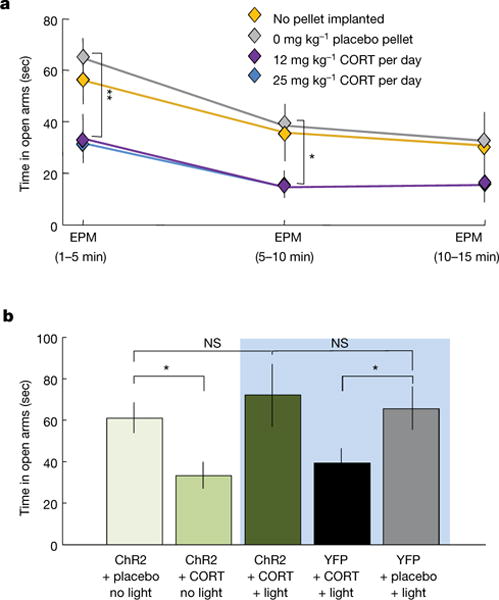
vmPFC-BMA projection reverses anxiogenic effect of corticosterone **a**, Chronic corticosterone (CORT) treatment decreased open-arm exploration. *n* = 10 no-pellet, 10 placebo pellet, 7 (12 mg kg^−1^) CORT, 8 (25 mg kg^−1^) CORT mice. **b**, vmPFC–amygdala activation reversed effect of CORT. NS, not significant. *n* = 15 YFP placebo + light, 15 YFP CORT + light, 15 ChR2 CORT + light, 7 ChR2 CORT + no light, 8 ChR2 placebo + no light; *P < 0.05, **P < 0.01, two-sided Wilcoxon test. Error bars, mean ± s.e.m.

Anxiolysis by the vmPFC–BMA circuit raised the possibility of top-down modulation of behaviour elicited by pathological conditions (such as high levels of corticosterone, since elevated cortisol-related stress hormones are known to be associated with anxiety disorders^38–40^). Indeed, chronic corticosterone treatment increased^41,42^ avoidance of open arms (Fig. 6a, P = 0.018, Wilcoxon rank-sum test), an effect reversed by vmPFC–amygdala activation (Fig. 6b).

## Discussion

Here we identify the BMA as the direct target of vmPFC that suppresses fear-related freezing and anxiety states. Although these and other regions have been studied separately, a precise direct target for well-resolved vmPFC projections in top-down control of anxiety and fear had not been identified, nor had roles for the vmPFC–BMA projection been demonstrated. We find that this pathway is sufficient and necessary for anxiolysis; that cells inhibited by aversive stimuli are over-represented in BMA, and that BMA activity suppresses freezing and elevated-anxiety states. We also show the major source of prefrontal input to ITCs is not vmPFC, but rather dmPFC; for such circuit-level insight, precise projection-validated targeting of vmPFC versus dmPFC was crucial (Supplementary Note 1).

While vmPFC-BMA activation suppressed freezing and elevated-anxiety states, neither bulk vmPFC activation (Extended Data Fig. 2a–c), nor control of other cortical (dmPFC) afferents to the target (amygdala; Fig. 1e–h), gave rise to similar effects. The lack of anxiolysis following direct vmPFC activation points to diversity in the vmPFC population, which probably includes cells that project to regions counteracting anxiolytic effects of vmPFC-BMA projections (Supplementary Note 4). Interestingly, inhibition of the vmPFC-amygdala projection increased respiration but not heart rate, as seen in the bed nucleus of the stria terminalis (BNST)^24^, suggesting that heart rate may be regulated by other mechanisms perhaps related to direct sympathetic activation.

vmPFC also innervates the cortical and medial amygdala^9^; these structures may be activated in vmPFC-amygdala:ChR2 mice, and are not ruled out in regulating anxiolysis. Nevertheless, direct activation of BMA inhibits freezing. Interestingly, the BMA projects to several areas implicated in anxiolysis, such as the central lateral amygdala^43^, the vmPFC, and the BNST anterodorsal nucleus^43^, but not to directly adjacent anxiogenic regions, such as the dmPFC and the BNST oval nucleus (Extended Data Fig. 9k,l)^24,43^. Although freezing was not increased by direct optical inhibition of BMA cell bodies (Extended Data Fig. 9j), pharmacological inhibition of the BMA increases freezing^37^, and its activation inhibits social stress-induced cardiovascular responses^44^.

The minimal vmPFC–ITC connectivity observed in mice (Figs 3c and 4c) does not conflict with studies in rats showing increased ITC activity following electrical^45^ or pharmacological vmPFC activation^46^, in which no direct projections were tested (such effects could be implemented through indirect routes such as via dmPFC, which we find directly projects to ITCs (Figs 3f and 4f). Influence of vmPFC on ITCs is not ruled out (despite the low functional^47^ and anatomical^8^ connectivity between these regions), and it remains possible that vmPFC–BMA and vmPFC–ITC influences could play complementary roles in regulating distinct fear or anxiety phenotypes. Diverse local direct target possibilities for mediating top-down control of fear have been hypothesized, including ITCs or BLA^48^; here we directly identify top-down control anatomy, as activation of BMA-projecting vmPFC neurons inhibits freezing (Fig. 5c).

A final intriguing aspect is that while direct BMA activation increased extinction, the extinction memory did not persist (Fig. 5k), whereas driving the vmPFC–BMA projection gave rise to stable extinction (Fig. 2b). Moreover, vmPFC–BMA stimulation only reduced freezing with several fear-conditioned tone presentations (Fig. 2b), suggesting that pairing cue exposure with vmPFC–BMA activity induces lasting plasticity. Such plasticity in vmPFC projections for lasting effects on fear memory is potentially relevant to therapeutic strategies and to natural anxiety and fear regulation.

## METHODS

### Subjects: mice

Male and female C57BL/6 mice were group-housed in a reverse 12 h light/dark cycle. Mice were 8 to 12 weeks old at the time of viral infusion. Food and water were given *ad libitum.* Dopamine receptor D1a (Drd1a)-Cre transgenic mice (EY266) were obtained from GENSAT. Ai9 *lox*-td Tomato mice were purchased from JAX (line 007902). All experimental protocols were approved by the Stanford University Institutional Animal Care and Use Committee and were in accordance with the guidelines from the National Institutes of Health. Sample sizes were chosen based on previous behavioural optogenetics studies on anxiety, which typically use 6–10 mice per group^24^. No statistical methods were used to predetermine sample size. Behavioural assays were conducted with male mice only, while both male and female mice were used for anatomical tracing and *in vitro* electrophysiology assays.

### Subjects: rats

All data shown are from mice, except for Extended Data Fig. 5c, which shows data from rats. Wild-type male Sprague Dawley rats (250–400 g) were obtained from Charles River and were individually housed on a standard 12 h light/dark cycle and given food and water *ad libitum.*

### Viruses

All the adeno-associated virus (AAV) vectors used were serotyped with AAV5 coat proteins and packaged by the University of North Carolina Vector Core. All virus stock solutions were diluted to 2 × 10^12^ particles per ml. For ChR2 and YFP mice, AAV5:CamK2α:ChR2(H134R)-eYFP and AAV5:CamK2α:eYFP were infused, respectively. The CamK2α promoter was used to induce opsin expression preferentially in excitatory projection cells. AAV5-DIO-Ef1α -mCherry (used in the BMA of GAD2cre mice) and AAV5-DIO-Ef1α-ChR2(H134R)-YFP (used in vmPFC-amy:CAV-ChR2 mice) were injected at 8 × 10^12^ particles per ml. The maps for these viral vectors are available at http://www.optogenetics.org. Rabies-ΔG-GFP was provided by B. K. Lim and R. C. Malenka. CAV-Cre virus was obtained from The Institute of Molecular Genetics of Montpellie vector core (France), at 6.2 × 10^12^ particles per ml.

### Surgery: mice

Eight-week-old mice were anaesthetized with 1.5–3.0% isoflurane and placed in a stereotaxic apparatus (Kopf Instruments). A scalpel was used to open an incision along the midline to expose the skull. After performing a craniotomy, 1.0 μl of AAV5-CamK2α-ChR2(H134R)-YFP at a titre of 2 × 10^12^ particles per ml was injected per site (vmPFC or dmPFC) using a 10 μ l nanofill syringe (World Precision Instruments) at 0.1 μl min^−1^. The syringe was coupled to a 33 gauge bevelled needle, and the bevel was placed to face the anterior side of the animal. The syringe was slowly retracted 20 min after the start of the infusion. A slow infusion rate followed by 10 min of waiting before retracting the syringe is crucial to restrict viral expression to the vmPFC or dmPFC. Mice in vmPFC and dmPFC groups received unilateral viral infusion and fibre optic cannula implantation (0.22 NA, 200 μm diameter; Doric Lenses) in either the vmPFC or the dmPFC. Infusion coordinates were: anteroposterior, 1.7 mm; mediolateral, 0.25 mm; dorsoventral, 3.3 mm for the vmPFC (injection centered in the dorsal peduncular cortex) and anteroposterior, + 1.9 mm; mediolateral, 0.25 mm; dorsoventral, 2.0 mm for the dmPFC. Fibre optic cannula implantation coordinates were: anteroposterior, 1.7 mm; mediolateral, 0.25 mm; drosoventral, 2.8 mm for the vmPFC and anteroposterior, +1.9 mm; mediolateral, 0.25 mm; dorsoventral, 1.5 mm for the dmPFC. For vmPFC–amygdala and dmPFC–amygdala mice, mPFC virus infusions were done bilaterally and fibreoptic cannulae were implanted bilaterally above amygdala (anteroposterior, −1.3 mm; mediolateral, 3.3 mm; dorsoventral, 4.7 mm). All coordinates were measured from bregma.

Viral infusions targeting the vmPFC were centred in the dorsal peduncular cortex (Supplementary Note 1). These injections produced strong opsin expression in the peduncular cortex and the adjacent infralimbic cortex. On average, 61 ± 14% of vmPFC cells expressed YFP (data not shown). Viral infusions targeting the dmPFC were centred in the cingulate cortex. These injections produced strong opsin expression in the cingulate cortex and the adjacent prelimbic cortex. Only mice with opsin expression restricted to either the vmPFC or the dmPFC were used for behavioural assays. Mice with proper injections did not have a substantial number of YFP-expressing cell bodies outside the target region. However, fibres may be visible outside the target region, as the vmPFC and the dmPFC are reciprocally connected^9^. The boundary between dmPFC and vmPFC was determined by comparing which coronal section in a reference brain atlas most closely corresponds to section being imaged. The boundaries were defined based on the position of easily identified structural landmarks, such as the forceps minor of the corpus callosum. Additionally, high magnification confocal imaging (20 ×) was used to evaluate if YFP-expressing cell bodies was contained in a specific mPFC subregion. The projection patterns from dmPFC and vmPFC-injected mice also differed. Specifically, fibres from the vmPFC were seen in the dorsomedial and posterior nuclei of the hypothalamus, but not in the ventromedial hypothalamus. Fibres from the dmPFC were not found to innervate any part of the hypothalamus noticeably. This pattern of hypothalamic projections is in good agreement with prior anatomy studies comparing vmPFC and dmPFC projections^9^. Thus, mice injected in the vmPFC showed prominent fibres in the BMA and hypothalamus, but not in the BLA, while mice injected in the dmPFC had the complementary projection pattern. All mice included in the study had both YFP-expressing cell bodies confined to an mPFC subregion and the expected fibre projection pattern.

For mice expressing ChR2-YFP, NpHR-YFP or YFP in the BMA, 0.5 μl of AAV5-CaMK2α∷ChR2(H134R)-YFP or AAV5-CaMK2α∷eNpHR3.0-YFP at a titre of 2 × 10^12^ particles per ml was injected in the BMA (anteroposterior, − 1.3 mm; mediolateral, 2.8 mm; dorsoventral, 5.6 mm from bregma), and fibre optic cannulae were implanted 0.5 mm above the infusion site. Adhesive cement (C&B metabond) and dental cement (Stoelting) were used to securely attach the fibre optic cannulae to the skull. For experiments employing CAV-Cre, this virus was injected at 6.2 × 10^12^ particles per ml in the BMA (0.5 μl bilaterally). We also injected 1.0 μl bilaterally of AAV5-DIO-Ef1α-ChR2-YFP (8 × 10^12^ particles per ml titre) in the vmPFC in these mice. For patch-clamping experiments using GAD2-Cre mice, we injected 1.0 μl of AAV5-Ef1α-mCherry (8 × 10^12^ particles per ml titre) in the BMA to label GAD2-expressing BMA interneurons. In these mice we also injected 1.0 μl bilaterally of AAV5-CamK2α-ChR2-YFP (4 × 10^12^ particles per ml titre) in the vmPFC. Mice recovered from surgeries in a heated cage before returning to their home cage. Half the mice in each cage were randomly assigned to YFP or ChR2/NpHR groups.

### Surgery: rats

Stereotactic surgeries were with rats (8–10 weeks of age) under isoflurane anaesthesia (4% initially, maintained at 2–3%). Respiratory rate and absence of the tail pinch response were monitored regularly. The scalp was shaved and rats were placed in the stereotax. A heating pad was used to prevent hypothermia. Lactated Ringer’s solution (5 ml kg^−1^ subcutaneous), buprenorphine (0.05 mg kg^−1^ subcutaneous) and enrofloxacin (5 mg kg^−1^, subcutaneous) were administrated to rats. A midline incision was made to expose the skull and a craniotomy was performed unilaterally over the mPFC Virus injections were delivered with a 10 μl syringe and 33 gauge bevelled needle injected at 100 nl min^−1^ using an injection pump. A single 1 μl injection (AAV5-CKIIα-SSFO-eYFP at a titre of 2–4 × 10^12^ particles per ml) was made at the following stereotactic coordinate to target the vmPFC (infralimbic cortex): anterioposterior, + 2.7 mm; mediolateral, 0.5 mm; dorsoventral, 5.0 mm from bregma. Following injection, the injection needle was kept at the injection site for 10 min then slowly withdrawn. The skin was sutured and the rats recovered in a clean cage under a heated lamp. Rats were euthanized and perfused 5 months following surgery to allow strong YFP expression in vmPFC terminals in the amygdala.

### Rabies tracing

For rabies tracing experiments, 1 μl of stock rabies-ΔG-GFP solution (provided by B. K. Lim and R. C. Malenka) was injected in the BMA (anterioposterior, − 1.3; mediolateral, 2.7; dorsoventral, 5.6). Mice were sacrificed 6 days following surgery. Brain slices were then obtained as described below in ‘Histology’. GFP-expressing cells were then counted on a Leica TCS SP5 scanning laser confocal microscope. Values plotted represent averages from 12 brain slices (three slices from four different mice).

### Retrobead tracing

Red retrobeads (LumaFluor) were injected undiluted using a 10 μl nanofill syringe (World Precision Instruments) at 0.1 μl min^−1^. The syringe was coupled to a 33 gauge bevelled needle, and the bevel was placed to face the anterior side of the animal. The syringe was slowly retracted 20 min after the start of the infusion.

### Light delivery

For ChR2 mice, blue light was generated by a 473 nm laser (Omicron Laserage) at 10 mW power to stimulate fibres in the amygdala and at 1 mW to activate mPFC cell bodies. Yellow light was generated by a 593.5 nm DPSS laser (MGL-F593.5; OEM Laser Systems), and bilaterally delivered to mice at 10 mW. A Master-8 pulse generator (A.M.P.I.) was used to drive the laser at 10 Hz, with 5-milisecond pulses for the entire duration of the tone (20 s). The laser output was delivered to the animal via an optical fibre (200 μm core, 0.22 numerical aperture, Doric Lenses), which was coupled to the fibre optic implanted on the animals through a zirconia sleeve.

### Elevated plus maze (EPM) and open field testing

Experiments were performed 3 weeks after surgery for vmPFC:ChR2 and BMA:ChR2 groups and 3 months after surgery for vmPFC-amy:ChR2, vmPFC-amy:NpHR and dmPFC-amy:ChR2 groups, to allow for sufficient opsin expression. Mice were handled for 3 days before behavioural testing. On the day of the experiment, the fibre optic cannulae implanted on animals were connected to a patch cord. The animal was allowed to recover from this handling for 1–5 min in a cage before behavioural testing. The elevated plus maze was made of plastic painted grey (Med Associates), and the open field was a custom-made box with white plastic walls (dimensions: 50 × 50 × 50 cm). In the beginning of the test mice were gently placed in the periphery of the open field or the closed arms of the EPM. The EPM and open field sessions lasted 15 and 20 min, respectively. The only exception is for the experiment with corticosterone-treated mice, which explored the EPM for only 5 min. For both paradigms the session was divided into blocks of 5 min, which started with an ‘off’ epoch and then alternated between ‘off’ and ‘on’ epochs. Laser light was delivered for 5-min during ‘on’ epochs only. Both opsin and YFP-expressing animals that spent less than 5% time exploring the open arms of the EPM or centre of the open field were excluded from assays in which the optogenetic manipulation was expected to increase anxiety, as these animals already had ceiling levels of anxiety at baseline. This exclusion criterion was pre-established before the start of the experiment. In the experiment in which mice were treated chronically with corticosterone, mice ran the EPM for only 5 min, and blue light was delivered during the entire session to all groups of mice as 10-Hz trains of 5-ms pulses. For all anxiety assays animal position tracking, behavioural scoring and velocity measurements were done by the Biobserve software automatically. All plots showing overall velocity were from open field exploration sessions. The experimenter was blind to the opsin group of the animal while running the experiment.

### Chronic treatment with corticosterone

Mice were anaesthetized with 1.5–3.0% isoflurane and a small incision was made on the side of the neck. Corticosterone or placebo pellets (#G-111, Innovative Research of America) were placed in the neck incision, and were positioned between the skin and the underlying muscle tissue. Behavioural experiments were performed 15 days following implantation of the pellets. We tested pellets containing 7.5 and 15 mg of corticosterone, which are active over 21 days. For a 30 g mouse these pellets correspond to doses of 12.5 and 25 mg kg^−1^ corticosterone per day. The pellets are made of a biodegradable matrix of cholesterol and cellulose, and allow for constant release of corticosterone.

### Fear conditioning

All mice were handled for three days before behavioural assays for 5 min per day. The pulse-generator driving the laser was synchronized to tone onset by coupling the laser pulse-generator to the tone with a TTL pulse originating from the fear conditioning software (Freeze Frame, Actimetrics). For cued fear conditioning experiments, mice were placed in a chamber with a grid floor connected to a shock generator (Coulbourn). Two minutes after being placed in the chamber, mice were exposed to 20-s tones (2.9 kHz, 85 dB) co-terminating with a foot shock at pseudo-random inter-trial intervals (~2 min average). The protocol in Fig. 2b used six tone-shock pairings (0.7 mA, 2-s shocks), and the experiments in Fig. 2a used four tone-shock pairings (0.4 mA, 1-s shocks). The chamber was cleaned with 70% ethanol at the end of each trial. The next day (day 2, fear extinction), mice were placed in a different context (floor and walls were changed and white vinegar was sprayed in the chamber). First, mice were exposed to one tone without light delivery, to measure fear retrieval (tone 1 on day 2). Subsequently, mice were exposed to 10 tones (tones 2–11 on day 2) to undergo fear extinction with paired blue light delivery (10 Hz train of 5-ms pulses for 20 s). On day 3 mice were placed in the same environment as in day 2 and were exposed to one presentation of the tone. For the strong contextual fear conditioning protocol shown in Fig. 2c, d, mice explored the fear conditioning chamber for 2 min. Subsequently, mice received five 1-s foot shocks (0.4 mA) with ~2 min intervals on day 1. On day 2 blue light was delivered bilaterally to the amygdala while mice explored the same environment and on day 3 mice were tested for extinction retrieval in the same environment. The mouse explored the environment for 5 min during days 2 and 3. The weaker contextual conditioning protocol shown in Fig. 2e, f was similar, except that mice were conditioned to three 0.4 mA 1-s shocks and exposed to the training environment for 20 min on day 2 with blue light delivered for the entire 20 min. The experimenter was blind to the opsin group of the animal while running the experiment.

### Respiratory and heart rate measurements

Respiratory rate and heart rate were measured using a pulse oximeter (MouseOx Plus; Starr Life Sciences). Pulse oximetry works by placing a collar clip around the mouse’s shaved neck. One side of the collar emits red and infrared light, which pass through the neck and is measured on the other side of the neck by a detector. Oxygen-bound haemoglobin and de-oxygenated haemoglobin respectively absorb the infrared and red light preferentially. The ratio of these absorbances can be used to calculate oxygen saturation. Additionally, the small dilations in blood vessels induced by heart beats and respiration can also be detected by the change in the absorbances of these wavelengths. The functioning and limitations of this technique have been described in detail elsewhere^49^.

Data was collected on a computer running MouseOx Plus software. Mice were shaved around the neck and acclimated to moving with the collar sensor used to by the pulse oximeter for three days. Additionally, mice were handled for three days before experimenting. Respiratory rate was recorded as a moving average of ten measurements recorded at 1 Hz. Heart rate was recorded as a moving average of five heart beats. Baseline respiratory rate was typically in the 160 to 230 breaths per min range, while heart rate was in the 690 to 790 beats per min range. The MouseOx Plus software generates errors during abrupt movements made by the animal, as these motion artefacts generate unreliable readings. Only error-free data was used from sessions in which the recordings had at least 30% of error-free samples. Recordings were obtained for either 15 min only in the home cage or for 5 min in the home cage and 10 min in the open field. The experimenter was blind to the opsin group of the animal while running the experiment.

### Microdrive construction and implantation

Microdrives were custom built using Neuralynx (Neuralynx) a 16-channel electronic interface board (EIB-16), as described elsewhere^34^. Stereotrodes were made with 25-μm Formvar-coated tungsten microwires (M165260, California Fine Wires) and were inserted into a metal cannula (GHX-24, Component Supply Co.), which in turn was attached to the ‘R’ channel slot of the EIB-16. A fibre optic cannula was glued to the outer part of the metal cannula, and glued to the protruding shaft of the stereotrode bundle, with care being taken not to glue the electrode tips. The stereotrodes were cut so that they extended 0.5 mm beyond than the end of the tip of the fibre optic. This design allows for light delivered through the cannula to reach the cells recorded by the stereotrode tips without having the fibre optic damage the cells being recorded. The EIB-16 was secured with screws (SHCX-080-6; Small Parts) to a custom-built teflon platform. The platform and the EIB were in turn secured to teflon cuffs with screws, allowing the assembly containing the stereotrodes, EIB and fibre optic to be lowered by turning the screws after the teflon cuffs have been cemented to the animal’s skull as described previously^34^. Before steretactically implanting the microdrive, one screw was placed in the front of the skull to increase the cemented (Grip Dental Cement; Dentsply) assembly’s physical stability. Another screw was placed over the cerebellum to serve as an electrical ground and was soldered to the ground slot in the EIB (slot labelled ‘G’).

### *In vivo* electrophysiology data acquisition

Recordings were performed as described previously^34^. Mice recuperated from the implantation of the microdrive for one week. Animals were then handled for three days and habituated to moving with the tether connecting the microdrive to the recording system. Stereotrodes were advanced until at least four well-isolated single units were present. Mice were then placed in the EPM for 15 min. Recordings were made with a unity-gain head-stage preamplifier (HS-16, Neuralynx) coupled to a fine-wire cable. Spikes larger than 25 μV were band-pass filtered (600–6,000 Hz) and recorded at 32 kHz. Neural data were acquired on a personal computer running Neuralynx Cheetah software.

Animal position was tracked at 30 Hz using an overhead camera that detected two small LEDs attached to the head-stage. In experiments that used blue light to activate vmPFC terminals in the amygdala, the output of the laser pulse generator was recorded along with local spiking activity by the same recording amplifiers, allowing synchronous acquisition of neural data and laser onset and offset times. Blue light was delivered using the same parameters employed in other behavioural assays described above (10-Hz trains of 5-ms pulses at 10 mW power). At the end of the experiment current was passed through one of the stereotrode channels to produce an electrical lesion in the site of recording. Recordings were performed for 35 min. During minutes 0 to 5 recordings were obtained in the home cage. From minutes 5 to 25 the animal explored the elevated plus maze (EPM). Lastly, during minutes 25 to 35 the animal explored the light/dark test (LDT).

### Antidromic spike recording

Mice expressing ChR2 in vmPFC cell bodies (injections as for behaviour) were anaesthetized using 1–2% isoflurane. Stimulation light pulses were delivered to the BMA using the same optical fibre type, coordinates, and stimulation pulse parameters as in behavioural experiments (5-ms pulses at 10 Hz). We used higher light power (80 mW) to maximize the chance of antidromic activation. Multiunit activity was simultaneously recorded across 32 in 2 animals (total 64 sites) spanning the entire mPFC (mediolateral, 0.4 mm; anteroposterior, 1.6 mm; dorsoventral, 1.8–3.3 mm from bregma) using a silicon probe (A1x32-6mm-50-177-Z32, NeuroNexus). Signals from each recording site were amplified, digitized and digitally filtered (600–6,000 Hz) using a multichannel recording system (RZ5D, Tucker-Davis Technologies). Multiunit firing was assessed by thresholding raw traces at three standard deviations from baseline using custom analysis software (MATLAB, Mathworks). As a positive control, the stimulation optical fibre was retargeted to the recording location in mPFC using an angled approach to directly elicit local spiking.

### Analysis of neural data

#### Code availability

All custom-written MATLAB code is available upon request.

#### Elevated plus maze

Following recording, single units were clustered manually offline with the SpikeSort3D software (Neuralynx). Data were then imported into MATLAB using custom-written scripts. Peri-stimulus time histograms (PSTHs) of spiking activity were constructed for a 100 ms time window centred at the onset of 5-ms laser pulses. EPM scores were computed for all recorded single units to estimate the extent to which each unit encoded arm type in the EPM^24,34^. The calculation of EPM scores has been explained in great detail in step-by-step fashion previously^24^. In brief fold-firing rates relative to the unit’s mean firing rate were calculated for each of the EPM’s compartments, and the score was computed by the formula

$$\begin{array}{l} \text{EPM Score}=\left(A-B\right)/\left(A+B\right),\;\text{where}\\ A=0.25\times \left(\left|\text{FL}-\text{FU}\right|+\left|\text{FL}-\text{FD}\right|+\left|\text{FR}-\text{FU}\right|+\left|\text{FR}-\text{FD}\right|\right)\;\text{and}\\ B=0.5\times \left(\left|\text{FL}-\text{FR}\right|+\left|\text{FU}-\text{FD}\right|\right) \end{array}$$

FL, FR, FU and FD are the fold-firing rate in left, right, up and down arms, respectively. For example, if the mean firing rate in the whole maze is 4 Hz, and the unit fired at 8 Hz in the left arm, then FL = 8/4 = 2. Left and right arms are closed arms, and up and down arms are open arms.

‘A’ is the mean difference in normalized firing rate between arms of different types, while ‘B’ is the mean difference for arms of the same type. The maximum score of 1 indicates that all differences in firing between different arms can be explained by arm type (B = 0, where a unit has identical high rates only closed arms or only in open arms). A high EPM score indicates that a unit fires preferentially either in both closed arms or in both open arms. A score of zero indicates that a single unit has the same firing rate in all compartments of the EPM. A negative score would be assigned to a single unit that has similar firing rates in arms of different types (for example, a unit has high rates in one of the open arms and one of the closed arms).

Cells with EPM scores equal or smaller than zero were classified as not task-related. The remaining cells (all of which have EPM scores > 0) were classified as either closed- or open-arm-preferring, if the cell displayed higher rates in the closed or open arms, respectively.

To calculate if the population of experimentally observed EPM scores was significantly different than expected by chance, a simulated distribution of scores was generated. For each unit with *n* spikes, 500 simulated scores were generated by calculating the EPM score of *n* randomly chosen timestamps 500 times. This artificially generated population of EPM scores was then compared to the scores obtained from actual BMA single units using Wilcoxon’s Rank sum test.

In order to analyse the stability of firing patterns of BMA cells in the EPM we calculated firing rates in the closed and open arms for the first and second halves of the EPM exploration session separately. We only used data from cells that fired at least 10 spikes in each arm type in each of the two halves of the session. EPM scores and preference of firing in a specific arm type were not significantly correlated with action potential waveform features that are commonly used to classify cells as excitatory or inhibitory (Supplementary Note 5).

#### Cued fear conditioning

Cells were classified as tone responsive if their firing rate during the tone presentation period was significantly different *(P <* 0.05 with the Wilcoxon rank sum test) from firing during baseline (the 40 s preceding the tone presentation).

### *In vitro* electrophysiology

Three months after injections, acute 300-μm coronal slices were prepared by transcardially perfusing the mice with an ice-cold sucrose solution (containing in mM: 125 NaCl, 2.5 KCl, 0.1 CaCl_2_, 3.9 MgCl_2_, 26 NaHCO_3_, 1.25 NaH_2_PO_4_-H_2_O, 2.5 glucose, 50 sucrose) and slicing the brain tissue in the same ice-cold sucrose solution using a vibratome (VT1200S, Leica). Slices containing the PFC were fixed in 4% paraformaldehyde and saved for verification of the ChR2 injection site. Slices containing the amygdala were allowed to recover for 1 h at 33 °C in artificial cerebrospinal fluid (aCSF; containing in mM: 125 NaCl, 2.5 KCl, 2 CaCl_2_, 1 MgCl_2_, 26 NaHCO_3_, 1.25 NaH_2_PO_4_-H_2_O, 11 glucose) bubbled with 95%O_2_/5%CO_2_. Whole-cell patch-clamp recordings were performed in the same aCSF solution at 30–32 °C. Where indicated, TTX (1 μm) and 4-AP (100 μM) were added to the aCSF. Resistance of the patch pipettes was 2.5–4 MΩ when filled with intracellular solution containing the following (in mM): 120 CsMeSO_3_,15 CsCl, 8 NaCl, 0.2 EGTA, 10 HEPES, 2 Mg-ATP, 0.3 Na-GTP, 10 TEA (tetraethylammonium), 5 QX-314 (lidocaine *N*-ethyl bromide), adjusted to pH 7.25 with CsOH. In some experiments, 0.2% biocytin was also included in the intracellular solution. Slices containing biocytin-filled cells were later processed as explained in ‘Immunocytochemistry Biocytin’, to allow accurate visualization of the recorded cell’s location. Signals were amplified with a Multiclamp 700B amplifier, acquired using a Digidata 1440A digitizer, sampled at 10 kHz, and filtered at 2 kHz. All data acquisition and analysis were performed using pCLAMP software (Molecular Devices). Neurons were visually identified for patching using an upright microscope (Olympus BX51WI) equipped with DIC optics, filter sets for visualizing YFP and tdTomato, and a CCD camera (RoleraXR, Q-Imaging). After break-in, neurons were voltage-clamped at − 65 mV. As neurons were recorded, pictures of the placement of the recording electrode were taken under low magnification (5 × /0.15 air objective) and the location of the recording was noted on a schematic of the amygdala. To stimulate ChR2 expressed in axon terminals from the PFC, 5-ms blue light pulses (~10 mW mm^−2^) were generated using a Spectra X LED light engine (Lumencor) and delivered to the slice via a 40 ×/0.8 water-immersion objective focused onto the recorded neuron. Pulses were delivered once every 30 s. Reponses sizes were calculated by baseline- subtracting and averaging 5–15 traces together, then calculating the peak amplitude in a 50 ms window after the light pulse. Neurons that did not show a peak amplitude in this window that exceeded 5 s.d. of the baseline noise were counted as non-responders. Recordings shown in Fig. 4 were performed in mice injected with 1 μl of 2 × 10^12^ titre AAV5-K2α CamK2α -ChR2-YFP virus, similar to the infusions done in vmPFC–amygdala:ChR2 mice. Infusions done in GAD2-Cre mice were done with 4 × 10^12^ titre (Extended Data Fig. 7g). This difference is probably why a higher fraction of BMA neurons were responsive to optical stimulation of vmPFC terminal in the latter experiment (91%) compared to Fig. 4 (63%). Importantly, cells from all three amygdala regions studied (BMA, BLA and ITC) were recorded from each mouse. This approach ensures that the unresponsiveness of ITC and BLA cells in mice expressing ChR2 in the vmPFC is not due to insufficient opsin expression or any other technical problem, as responsive BMA cells were recorded from the same animal.

### Histology

Mice were deeply anaesthetized and transcardially perfused with ice-cold 4% paraformaldehyde in PBS. Brains were fixed overnight in 4% paraformaldehyde and then equilibrated in 30% sucrose in PBS. Brains were sliced in 40-μm-thick coronal slices in a freezing microtome, and stored in cryoprotectant solution (a mixture of 5:6:9 volumes of glycerol, ethylene glycol and PBS) at 4 °C until being mounted on slides on PVA-DABCO. Nuclei were stained by incubating with DAPI (1:50,000 in PBS, 25 min). In animals with retrobead injections brain sections were stored in PBS at 4 °C in Fluoromount-G (Southern Biotech). Confocal images were obtained on a Leica TCS SP5 scanning laser microscope using a 20 ×/0.70 numerical aperture oil immersion objective. Only mice with opsin expression restricted to the target region and correct fibre optic cannula placement were used.

### Immunocytochemistry and c-Fos counting: c-Fos

vmPFC–amygdala: ChR2 mice received blue light in the amygdala (10 Hz, 5-ms pulses, 10 mW power) for 10 min. Animals were perfused 90 min after receiving optical stimulation. Following perfusion, brain slices were obtained as described above in ‘Histology’. c-Fos staining was performed as described previously^46^. In brief, coronal sections containing the amygdala were washed in PBS (three 10-min washes), and were then incubated for 1 h in blocking solution with 0.2% Triton-X-100 and 2% normal donkey serum. Sections were then incubated overnight with anti c-Fos primary antibody (rabbit anti c-Fos 1:500, Cell Signaling Technology, cat. no. 2250S). The next day the sections were washed in PBS (three 10-min washes) and incubated at 1 h at room temperature with the secondary antibody at 1:500 dilution (donkey anti-rabbit antibody conjugated to Cy3, Jackson Laboratories). Slices were washed three times in PBS and mounted on slides with PVA-Dabco (Sigma). c-Fos counting was performed blinded to treatment group on a Leica TCS SP5 scanning laser confocal microscope. To stain cells against GABA the same procedure was employed, but this time using anti-GABA antibody (Sigma, cat. no. A2052).

### Immunocytochemistry and c-Fos counting: FoxP2

Staining against FoxP2 was done similarly to the procedure described above for c-Fos, but with the alterations described below. The first incubation was in 0.3% Triton-X in 3% normal donkey serum, and it lasted 2 h. The primary antibody used was anti-FoxP2 made in Rabbit (AbCam, cat. no.16046) at 1:500 dilution for 12 h at 4 °C. The secondary antibody was anti-rabbit conjugated to Alexa Fluor 647 (AbCam, cat. no. 150075), incubated at 1:500 dilution for 3 h at room temperature.

### Immunocytochemistry and c-Fos counting: GABA

Same procedure as FoxP2 staining, except that anti-GABA antibody was used (Sigma, cat. no. A2052), with 0.1% Triton-X.

### Immunocytochemistry and c-Fos counting: biocytin

The same procedure used to stain against c-Fos was employed to visualize cells filled with biocytin during patch clamping recordings, with the following modifications. The first incubation was in 0.3% Triton-X, and 3% normal donkey serum, and it lasted for 2 h. Instead of an incubation with a secondary antibody sections were incubated with streptavidin conjugated to Alexa Fluor 647 (5 μg ml^−1^) for 2 h at room temperature.

### CLARITY

Slices from vmPFC-amygdala:YFP mice were clarified using the CLARITY procedure as explained elsewhere^50^. In brief, after patching, 300-μm sections were fixed in hydrogel solution (4% PFA, 1% acrylamide w/Bis) for 20 h at 4 °C. After polymerization (37 °C, 4 h), the sections were clarified in 4% SDS for 2 days (at 37 °C). The cleared sections were washed three times with PBST (0.1% TritonX) for a total of 4 h at room temperature. Alexa647-conjugated anti-GFP antibody (1:100, Invitrogen) was used to stain YFP (room temperature, overnight). Stained sections were incubated in FocusClear (CelExplorer Labs Co.) for 1 h before imaging with a confocal microscope.

### Statistics

Two-way repeated measures ANOVA was used. Wilcoxon rank sum post hoc tests were used following the ANOVA only if significant main or interaction effects were detected. Two-tailed tests were used throughout with α = 0.05. The Wilcoxon test is non-parametric, and as such it does not require the data to be normally distributed. Nevertheless, we tested the normality of all the behavioural data being used with the Lilliefors test, and found that our behavioural data passed this test. The variances of the groups being compared were not significantly different in any of the comparisons. Variance equality was tested using the *F*-test. Asterisks in the figures indicate the *P* values for the post hoc test at a given epoch. Standard error of the mean (s.e.m.) was plotted in each figure as an estimate of variation within each group of data. Multiple comparisons were adjusted with the false discovery rate method. Our experimental design of having light stimulation ‘on’ and ‘off’ epochs in a single session for each animal allows us to use repeated measures ANOVA to test if light delivery significantly alters the behaviour of interest relative to its own baseline (that is, the first light off epoch). This strategy is statistically more powerful than comparing YFP and ChR2 groups with an unpaired test. As this statistical test evaluates changes relative to baseline, we opted to plot normalized data. In this way the data presentation accurately represents the differences that are being statistically tested. Importantly, there were no significant differences at baseline between YFP and experimental groups in behavioural or physiological measures of anxiety. In all figure legends *n* refers to biological replicates.

## Extended Data

**Extended Data Table 1:** Anxiety behavioural data in absolute values

	Figure location	0–5 min.	5–10 min.	10–15 min.	15–20 min.
vmPFC-amy:YFP EPM	1b	57±12	73±15	63±19	
vmPFC-amy:ChR2 EPM	1b	63±14	50±11	61±14	
dmPFC-amy:YFP EPM	1f	53±7	48±17	38±5	
dmPFC-amy:ChR2 EPM	1f	50±12	46±16	42±17	
BMA:YFP EPM	5j	64±14	35±11	31±14	
BMA:ChR2 EPM	5j	65±19	77±23	45±19	
BMA:YFP OFT	5l	62±9.6	84±13	91±17	122±23
BMA:NpHR OFT	5l	66±8.5	66±10	73±13	71±9.5

Table displaying time spent (in seconds) in the open arms of the elevated plus maze (EPM) or the centre of the open field test (OFT). Values are expressed as averages ± s.e.m.

## Supplementary Material

Movie
